# Von der Rekonstruktion zur Funktion: Tympanoplastik-Training mit Real-Time-Feedback

**DOI:** 10.1007/s00106-020-00941-x

**Published:** 2020-09-10

**Authors:** T. Beleites, T. Zahnert, M.-L. Polk, A. Kluge, M. Neudert, M. Kemper

**Affiliations:** Klinik und Poliklinik für HNO, TU Dresden, Universitätsklinikum, Fetscherstr. 74, 01307 Dresden, Deutschland

**Keywords:** Ohrchirurgie, Trommelfell, Gehörknöchelchen, Ossikelprothese, Mittelohr, Ear surgery, Eardrum, Ear ossicles, Ossicular prosthesis, Middle ear

## Abstract

**Hintergrund:**

In der Mittelohrchirurgie bedarf es ausgezeichneter feinmotorischer Fertigkeiten. Aufgrund des hohen Komplikationspotenzials im Mittelohr ist die Ausbildung dieser Fertigkeiten am Modell anzustreben.

**Fragestellung:**

Wie gut ist die Ausbildungsmöglichkeit an geeigneten Modellen? Können die am Modell erlernten Fertigkeiten in die intraoperative Situation übertragen werden? Beeinflusst das Modell und die Ausbildung daran die zukünftige Ohrchirurgie?

**Material und Methode:**

Vorliegende Publikationen und eigene Erfahrungen am Dresdener Tympanoplastikmodell (DTM) wurden analysiert und diskutiert.

**Ergebnisse:**

Obwohl die Mittelohrchirurgie hohe Anforderungen an den Ausführenden stellt und am Sinnesorgan Ohr schwerwiegende Komplikationen drohen, gibt es bisher nur wenige Trainingsmöglichkeiten dafür. Das DTM ist ein validiertes Übungsmodell, das diese Lücke schließen kann. Durch eine Real-Time-Feedback-Variante des Modells kann auch das Verständnis für Rekonstruktionsqualität und intraoperative akustische Noxen verbessert werden. Die Übertragung des Real-Time-Feedback-Gedankens in die reale Mittelohrchirurgie kann die Rekonstruktionsqualität zukünftig verbessern.

**Schlussfolgerungen:**

Das Training an geeigneten Modellen ist speziell beim Erlernen der Mittelohrchirurgie anzustreben. Mit dem Real-Time-Feedback kann beim Lernen und Operieren eine weitere Sinneswahrnehmung in die eigene und fremde Qualitätskontrolle der Tympanoplastik sehr wirksam einbezogen werden.

## Abstract (en)

**Background:**

Surgery of the middle ear requires a very high level of fine motor skills. Due to the increased potential for complications in middle ear operations, it is appropriate to acquire these skills beforehand by operating on a model.

**Objective:**

How satisfactory is the training on suitable models? Are the skills acquired from working on a model transferable to intraoperative situations? Will the type of model and its use for training influence ear surgery in the future?

**Material and methods:**

Available publications and own experiences with the Dresden tympanoplasty model (DTM) are analyzed and discussed.

**Results:**

Although middle ear surgery makes very high demands on the surgeon and there is a significant risk for severe complications to the ear as a sense organ, there are currently very few options to train surgeons in advance. The DTM is a validated training model, which is capable of closing this gap. Due to the possibility of using a real-time feedback variation of the model, the understanding for reconstruction quality and intraoperative acoustic stress can be improved. The translation of the real-time feedback idea into actual middle ear surgery can improve reconstruction quality in the future.

**Conclusion:**

Training on suitable models is indispensable, especially when training as a surgeon to carry out middle ear operations. Adding another sense perception to the internal and external quality assessment of tympanoplasty by inclusion of the real-time feedback option, can optimize learning and operating processes.

Unser Sinnesorgan für die Schallwahrnehmung ist auf kleinem Raum in festem Knochen eingebettet, hat durch die angrenzende mittlere und hintere Schädelgrube nur einen sehr begrenzten Zugang und ist wichtigen Strukturen wie N. facialis und A. carotis interna unmittelbar benachbart. Die Chirurgie des Ohrs erfordert bei komplexer Anatomie sicheres und geschicktes Agieren auf engstem Raum. Das Erlernen von Standardprozeduren der Mittelohrchirurgie am Patienten ist aufgrund gravierender Folgen für diesen bei fehlerhaftem Vorgehen problematisch. Zum risikofreien Training verschiedener Schritte der Ohroperation erscheint daher ein Trainingsmodell mit Real-Time-Feedback wünschenswert. Außerdem wird die Übertragung der Real-Time-Feedback-Methode in die reale Operationssituation diskutiert.

## Feinmotorische Präzision in der Mittelohrchirurgie

Bei der Mittelohrchirurgie werden 3 wesentliche Ziele verfolgt. An erster Stelle sollen gefährdende oder behindernde Prozesse wie Entzündungen, Neubildungen, Gewebsverdickungen oder Gewebsverlagerungen sicher entfernt werden. Das zweite Ziel ist die funktionell optimale Wiederherstellung des Schallleitungsapparats oder die implantatgestützte Rehabilitation des Hörvermögens. Als drittes Ziel kann die langzeitstabile Rekonstruktion funktionsrelevanter Begleitstrukturen wie Gehörgangswand, Tuba auditiva oder Tegmen tympani unter dem Aspekt einer zukünftigen Pflegeminimierung des Ohrs angesehen werden. Im Ergebnis der durchgeführten Mittelohrchirurgie soll ein gut hörfähiger und weitgehend vom HNO-Arzt unabhängiger Patient resultieren. Zur Erreichung dieser Ziele ist ein hochgradig präzises Arbeiten auf engstem Raum und ausgezeichnetes anatomisches Orientierungsvermögen erforderlich. Die präzise Führung der Instrumente kann eine Schädigung des Innenohrs weitestgehend vermeiden und ist für den erfolgreichen Aufbau der schallübertragenden Strukturen unabdingbar. Diese feinmotorische Präzision und eine ruhige Hand sind besonders bei Arbeiten am Stapes und dessen Umgebung erforderlich.

Neben der Entfernung von Cholesteatommatrix, narbigen Verwachsungen und entzündlich verändertem Gewebe erfordert auch der Wiederaufbau von Anteilen der Gehörknöchelchenkette viel Feingefühl und Übung. Defekte der Kette, meist bedingt durch chronische Entzündungsprozesse finden sich am häufigsten am langen Ambossschenkel [23]. Für die Art der Versorgung spielt der noch erhaltene Anteil dieses Knochenfortsatzes die entscheidende Rolle. Zahnert [25] empfiehlt bei kleinen Defekten des langen Ambossschenkels ohne Längenverlust eine Knochenspaninterposition, bei Längenverlust bis 30 % den Einsatz einer Winkelprothese oder die Verwendung von Knochenzement und bei weitergehendem Substanzverlust die Rekonstruktion mit dem individuell zurechtgeschliffenen Ambossrest, einer Partialprothese oder die Verwendung von Knochenzement. Wenn der Steigbügel in seiner Bogenstruktur geschädigt ist, meist in Kombination mit dem langen Ambossschenkel [23], ist für die Rekonstruktion der Kette eine *T*otal *O*ssicular *R*eplacement *P*rosthesis (TORP) erforderlich. Die zwischen der Stapesfußplatte und dem meist rekonstruierten Trommelfell interponierten Totalprothesen können bei optimaler Positionierung, gemessen unter Laborbedingungen, eine der gesunden Kette gleichwertige Übertragung erreichen [24]. Messungen der Schallübertragung nach Protheseneinsatz bei realen Patienten zeigen nach Ausheilung insbesondere nach TORP-Verwendung fortbestehende relevante Schallleitungsschwerhörigkeiten [5]. Diese Diskrepanz beruht auf einer suboptimalen intraoperativen Positionierung oder postoperativen Veränderungen des Mittelohrs einschließlich der Prothesendislokation. Um eine Dislokation im besonders gefährdeten Fußplattenbereich zu verhindern, kann zur Stabilisation ein Omega-Connector (Fa. Heinz Kurz, Dusslingen) [20] oder ein „Knorpelschuh“ [10] verwendet werden. Alle Maßnahmen an der intakten Ossikelkette oder dem Stapes haben in jedem Fall eine mehr oder weniger hohe Ringbandbelastung zur Folge und können bei unsachgemäßer Ausführung schnell zu Läsionen von Ringband oder Fußplatte führen. Eine derartige Schädigung birgt eine hohe Gefahr der Hörminderung bis zur Ertaubung des operierten Ohrs. Demnach ist die hochentwickelte feinmotorische Präzision insbesondere für den Erhalt und die Verbesserung der Hörfunktion von besonderer Bedeutung.

## Modellentwicklung für die ohrchirurgische Ausbildung

Der Berufsanfänger in der Hals‑, Nasen- und Ohrenheilkunde hat typischerweise sehr wenig Erfahrung in Bezug auf mikroskopisches Arbeiten mit grazilen Instrumenten auf sehr engem Raum. Der knöcherne Gehörgang schränkt als Zugang die Bewegungsfreiheit weiter ein, weswegen sich für die Mittelohrchirurgie der Begriff „Schlüssellochchirurgie“ etabliert hat. Möglichkeiten zum Erlernen dieser feinmotorischen Fertigkeiten gepaart mit der erforderlichen Geduld bei der Ausführung sind weder im Studium der Medizin noch in der Weiterbildung zum HNO-Facharzt reichhaltig vorhanden. Die Untersuchung des Ohrs, Manipulationen am Gehörgang und das Erlernen der Parazentese und Paukendrainage sind die einzigen relevanten Vorübungen für die Mittelohrchirurgie. Dementsprechend ist ein relativ langer Zeitabschnitt beim Erlernen der Ohrchirurgie der Zugangsdarstellung gewidmet, ohne dass Manipulationen an der Gehörknöchelchenkette durchgeführt werden. Begleitend werden Präparationskurse an Felsenbeinmodellen angeboten, die insbesondere das anatomische Verständnis des Felsenbeins befördern. Ideal ist ein klinikeigenes Felsenbeinlabor mit der Möglichkeit, an eigenen Präparaten zu arbeiten, aber diese Möglichkeit besteht nur an wenigen Kliniken.

**Figure 1:**
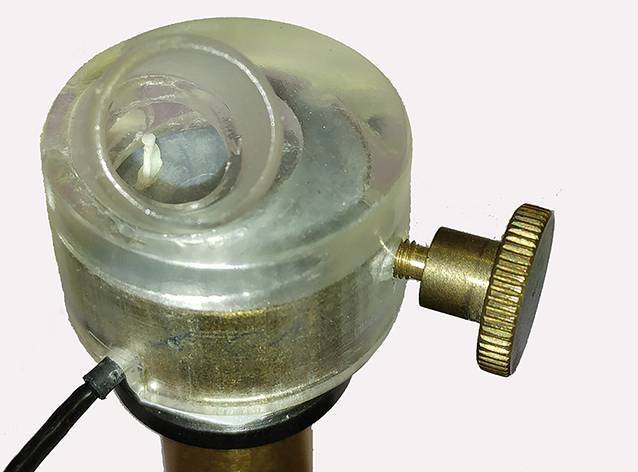

Selbstständig an den Ossikeln zu präparieren oder zu rekonstruieren, erfordert ob der drastischen Komplikationsmöglichkeiten und der fragilen Strukturen ein hohes Maß an Übung, Geduld und mechanischem Feingefühl. Zum Erlernen dieser Fertigkeiten bedarf es einer präzisen Anleitung und intensiven Begleitung durch den Lehrenden. Aus dieser Situation ergibt sich nahezu zwangsläufig der Bedarf für ein Übungsmodell zum Erlernen der Rekonstruktion von Trommelfell und Ossikelkette. Für die Stapeschirurgie wurde von Mathews bereits 1997 [14] ein einfach nachbaubares Modell aus Pappbecher und Holzstäbchen vorgestellt. Eine Bauanleitung zu einem Trainingsmodell aus Verbrauchsmaterial veröffentlichten Owa et al. 2003 [18]. Ein modulares Modell mit hohem pädagogischem Anspruch, aber aufwendiger Herstellung veröffentlichten Mills und Lee ebenfalls 2003 [15]. An diesem Modell konnte nach Einsetzen des Mittelohrmoduls auch eine Ossikuloplastik geübt werden. Das 2005 in der Dresdener HNO-Universitätsklinik entwickelte Tympanoplastikmodell sollte neben einer guten Verfügbarkeit durch kostengünstige Konstruktion auch realitätsnahe Übungsmöglichkeiten für die Rekonstruktion des Trommelfells und das Aufsetzen einer PORP (*P*artial *O*ssicular *R*eplacement *P*rosthesis) bieten (Abb. 1). Mit der breiteren Anwendung des 3‑D-Drucks wurden eine Reihe von realitätsnahen Felsenbeinmodellen vorgestellt. Neben der Möglichkeit zum Üben an kindlichen Dimensionen [13] wurde auch auf die realitätsnahen Materialeigenschaften [12] großer Wert gelegt. Ein ausgeklügeltes Modell mit simulierter Blutung im Operationsfeld und zu entfernendem Cholesteatom wurde 2015 von Dedmon [4] beschrieben.

## Erfahrungen mit dem Dresdener Tympanoplastikmodell

Bei der Anwendung des Dresdener Tympanoplastikmodells (DTM) wurde ein intensiver Betreuungsbedarf der Lernenden offenbar. Dabei ist es in erster Linie die Ergebnisqualität, die naturgemäß von den Lernenden nicht sicher eingeschätzt werden konnte,– der wichtigste Faktor zur Einordnung der geleisteten Arbeit. Durch eine unabhängige, möglichst objektive Bewertung des Ergebnisses kann der Übende feststellen, wie genau er die Aufgabenstellung erfasst und den Erwartungen entsprochen hat. Übertragen auf den Patienten entspricht das dem klinisch-anatomischen Operationsergebnis.

**Figure 2:**
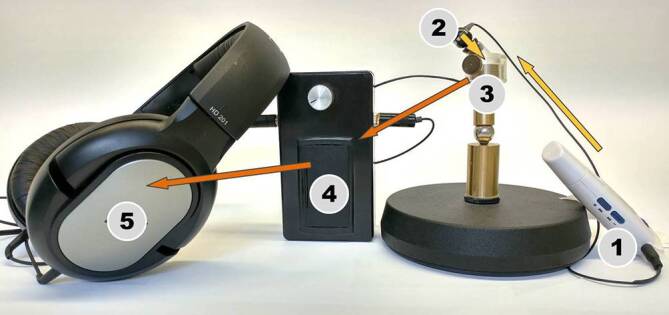

Für den Patienten selbst ist jedoch das funktionelle Ergebnis entscheidend. Für ein funktionell gutes Ergebnis muss neben dem optimalen Zusammenspiel der vorhandenen und rekonstruierten Anteile der Schallübertragung auch eine schadensfreie Präparation erfolgen. Um den Weg zum Ergebnis reflektieren zu können, ist eine Beobachtung aller Schritte und entsprechende Rückkopplung vom Lehrenden bei jedem Einzelnen erforderlich. Dies stellt einen enormen personellen Aufwand verbunden mit einer intensiven Kommunikation dar. Um allen Lernenden auch ohne diesen Aufwand die Qualität ihrer Arbeit widerzuspiegeln und die Konsequenzen des aktuellen Handelns bewusstzumachen, wurde ein Trainingsmodell mit Real-Time-Feedback entwickelt [7]. Dabei wurde aufbauend auf dem vorhandenen Design des DTM das Innenohr durch ein Mikrofon simuliert. Der auf den Stapes übertragene Schall inklusive aller instrumentellen Manipulationen an selbigem wurden über einen kleinen Verstärker und daran angeschlossene Kopfhörer den Ohren der Manipulierenden zugeleitet und damit sofort wahrnehmbar. Für die Ergebnisbeurteilung kann über den Ohrhörer einer elektronischen Musikquelle Musik in den künstlichen Gehörgang eingespielt und über den Kopfhörer beurteilt werden (Abb. 2). Mit dieser abschließenden Kontrolle kann die Qualität der Rekonstruktion gehört werden.

## Lehre am Modell

Das Dresdener Tympanoplastikmodell (DTM) wurde und wird in seiner ersten passiven Bauweise im Studentenunterricht und im Rahmen der internationalen Tympanoplastikkurse regelmäßig eingesetzt und evaluiert. An diesem Modell lassen sich die Dimensionen und anatomischen Lagebeziehungen auf einfache Weise nachvollziehen. Damit können 2 umschriebene Aufgaben gelöst werden:

- das Aufsetzen einer Partialprothese (PORP) und
- die Rekonstruktion eines kompletten Trommelfells.

Lernziel ist zunächst die Gewöhnung an die Manipulation mit dem Ohrinstrumentarium in einem auf die Dimensionen einer Paukenhöhle begrenzten Raum. In diesen Raum ist eine 3‑D-gedruckte Kopie eines realen Stapes elastisch eingeklebt. Dabei wurde ein Membranmaterial mit annähernd ringbandähnlichen elastischen Eigenschaften gewählt [7]. Damit ist ein deutlich vereinfachter mittelohrähnlicher Parcours geschaffen, der wesentliche Fertigkeiten zum Einsatz von PORP schulen kann. Die Rekonstruktion des Trommelfells ist im Vergleich zur realen intraoperativen Situation deutlich einfacher. Dennoch kann der Umgang mit und das Gefühl für dort eingesetzte Materialien trainiert werden. Die Autoren verwenden hierfür Knorpel und Perichondrium aus Schaf- oder Schweineohren, die tiefgefroren gut gelagert werden können.

## Evaluation des DTM

Um den Wert des Modells zu beschreiben, wurde es von den daran arbeitenden Studenten und erfahrenen Ohroperateuren evaluiert [2]. Bei dieser Evaluation wurde die anatomische Vergleichbarkeit des Modells von Anfängern deutlich besser bewertet, was auf die Vereinfachung hindeutet. Die aber dennoch gute Bewertung der anatomischen Entsprechung durch die erfahrenen Beurteiler dokumentiert ein, trotz der Einfachheit, insgesamt gelungenes Design. Von den 13 Fragen zur Verwendung des Modells wurde von den erfahrenen Ohroperateuren die Frage der Eignung zum Erlernen der Kettenrekonstruktion mit Abstand am besten bewertet. Diese insgesamt sehr positive Resonanz in verschiedenen Lehrsituationen war ausschlaggebend, das Design grundsätzlich auch für die Weiterentwicklung beizubehalten.

In einem 2. Schritt wurde der Lernerfolg von Studenten an diesem Modell untersucht [16]. Dafür wurden Medizinstudenten des 5. Studienjahrs randomisiert ausgewählt und in 2 Gruppen aufgeteilt. In einem Zeitraum von 21 Tagen wurde mit den Studenten der Übungsgruppe an den Tagen 1, 7 und 14 die Rekonstruktion von Trommelfell und Kette trainiert. Die Kontrollgruppe erhielt nur am Tag 1 die Möglichkeit, am Modell zu üben. Am Tag 21, dem Endpunkt der Untersuchung, wurden beide Gruppen in ihren Fähigkeiten am Modell bewertet. Im Ergebnis konnte ein signifikant besseres und schnelleres Rekonstruktionsergebnis der zwischendurch übenden Studenten im Vergleich zur Kontrollgruppe nachgewiesen werden. Außerdem konnte bei den häufiger am Modell trainierten Studenten eine signifikant höhere Motivation für die Ergreifung einer mikrochirurgischen Spezialisierung nach den 3 Wochen eruiert werden als in der Vergleichsgruppe. Auch die Freude bei der Arbeit am Modell wurde von den trainierten Studenten hinterher signifikant höher eingeschätzt. Gute Erfolge beim Erlernen der Paukendrainage mittels eines speziellen Modells [8] und beim modellbasiertem Training der endoskopischen Ohrchirurgie [1] unterstreichen den generellen Wert von Lehrmodellen in der ohrchirurgischen Ausbildung. Clark et al. haben einen weltweit hohen Bedarf modellbasierter Lehre in der Mittelohrchirurgie gesehen und zusätzlich Aufgabenstellungen und Schwerpunkte der Bewertung für ihr Modell formuliert [3], um eine einfache Nachahmung zu ermöglichen.

**Figure 3:**
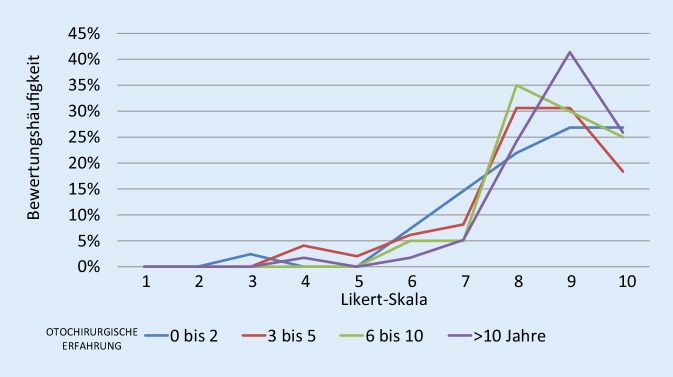

**Figure 4:**
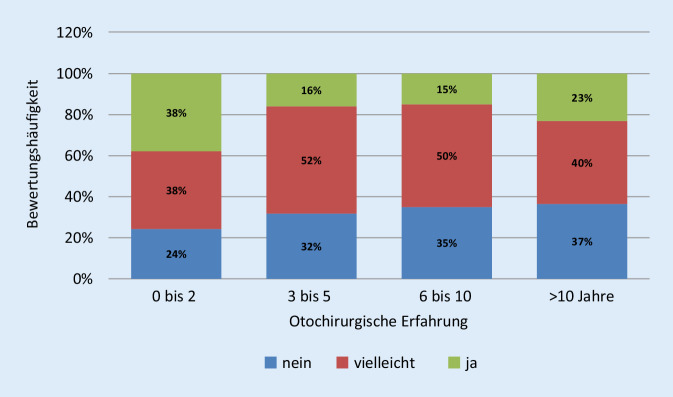

Bei dem weiterentwickelten DTM mit Real-Time-Feedback wurde auch eine Evaluation durch 214 internationalen Ohrchirurgen durchgeführt. Die bisher noch nicht veröffentlichten Ergebnisse zeigen ebenfalls eine durchweg positive Einschätzung des Modells sowohl in seiner Nähe zur Mittelohranatomie als auch in seiner Eignung zum Erlernen der Ohrchirurgie (Abb. 3). Die in der Anwendung durchweg als bedeutungsvoll eingestufte Rückkoppelung aller Manipulationen hat auch bei Ohroperateuren mit mehr als 10-jähriger Erfahrung Erstaunen über die akustische Belastung des Patienten während der Operation ausgelöst. Fast zwei Drittel aller Ohrchirurgen mit mehr als 10 Jahren Erfahrung wollten nach Verwendung des aktiven Feedbackmodells ihre Operationstechnik überdenken (Abb. 4).

Als hilfreich für das Verständnis der Schallübertragung wird darüber hinaus das Manipulationsfenster in diesem Modell angesehen. Hierbei kann nach vollständiger Rekonstruktion der Sitz der Prothese auf dem Stapesköpfchen verändert oder die Ankopplung der Prothese an das Trommelfell in der Spannung angepasst werden. Während dieser Manipulationen bleibt sowohl die Schalleinspielung über den Gehörgang als auch die Übertragung vom Modell auf den Kopfhörer erhalten. Entsprechend der vorgenommenen Manipulation ändert sich somit in Echtzeit der Höreindruck über den Kopfhörer.

## Operationsbegleitende Qualitätskontrolle

Insbesondere bei der Platzierung einer Prothese im Mittelohr muss ein hohes Maß der Kontrolle über die Kraftapplikation und das Bewegungsausmaß beim Operateur vorhanden sein. Die sofortige Messung und Rückkopplung dieser Parameter gibt dem Ohrchirurgen Aufschluss über die Qualität des Protheseneinsatzes und somit die Möglichkeit der gezielten Weiterentwicklung seiner feinmotorischen Fertigkeiten. So wurde von Nguyen et al. bei einer Qualitätskontrolle am Trainingsmodell für die Stapesplastik mit Kraftsensoren die effektive Verbesserung von feinmotorischen Fertigkeiten und operativer Technik nachgewiesen [17]. Die Anwendung der an Modellen gewonnenen Erfahrungen erfordern von dem jeweiligen Ohroperateur dennoch viel Flexibilität und Vorstellungsvermögen, da die Übertragung auf eine reale Rekonstruktion von individuellen anatomischen Bedingungen und wechselnden Operationsbedingungen wie entzündlich verdickter Schleimhaut, vorliegendem Cholesteatom, Kettenarrosion, Kettenversteifung oder intraoperativer Blutung erschwert wird. Das funktionelle Ergebnis der Rekonstruktion kann erst Wochen später beim abschließenden Audiogramm festgestellt werden. Eine Zuordnung der spezifischen intraoperativen Situation und des resultierenden audiologischen Ergebnisses zur Optimierung der Rekonstruktionstechnik ist in diesem Setting nur eingeschränkt vorstellbar. Ein intraoperatives Real-Time-Feedback zur Rekonstruktionsqualität, insbesondere bei der Ossikuloplastik, ist daher zukünftig wünschenswert. Neben der Optimierung des Ergebnisses bei jedem einzelnen Patienten sollte auch die Lernkurve des Operateurs durch eine solche Implementierung an Steilheit gewinnen.

Ein intraoperatives Real-Time-Feedback-System kann die Qualität der Schallübertragung messen und mit vorhandenen Parametern vergleichen. Durch die Rückmeldung der Messergebnisse an den Operateur ist eine sofortige Anpassung der Rekonstruktion durch den Operateur bis zum Erreichen eines individuellen Optimums möglich. Die Versorgungsqualität der operierten Patienten kann mit einem derartigen System nicht nur annehmbar verbessert werden, sondern ist hiermit auch objektiv zu dokumentieren. Im juristischen Streitfall kann eine solche Dokumentation die intraoperative Situation nachvollziehbar darstellen. Die Dokumentation des funktionellen Rekonstruktionsverlaufs und der Ergebnisqualität sollte die postoperative Kommunikation mit dem Patienten wesentlich anschaulicher gestalten. Es ist anzunehmen, dass durch eine gut nachvollziehbare Dokumentation der intraoperativen Übertragungsqualität die Akzeptanz des nicht immer als optimal empfundenen Operationsergebnisses beim Patienten erhöht und damit die Behandlungszufriedenheit insgesamt verbessert.

Der erste Schritt für die Entwicklung eines intraoperatives Real-Time-Feedback-Systems ist die Messung von schädigenden und gewünschten mechanischen Einflüssen auf das Innenohr. Der Einfluss von gefährdenden Faktoren, wie instrumentelle Präparation im Mittelohr, Bohrlärm oder Manipulation am Innenohr, aber auch die Qualität der Schallübertragung des rekonstruierten Mittelohrs müssen verlässlich eruiert werden können. Intraoperativ kann der resultierende Effekt am Innenohr berührungsfrei mit der Laser-Doppler-Vibrometrie (LDV) quantifiziert werden [9]. Die definierte Anregung der Kette über einen Lautsprecher im Gehörgang ist für die intraoperative Messung nur im Ausnahmefall möglich. Bei Fortsetzung der Operation während der Messung wird eine definierte akustische Quelle benötigt, die weder Bewegungsfreiheit noch die erforderliche Sterilität beeinträchtigt. In einer Machbarkeitsstudie zum intraoperativen Real-Time-Feedback wurde diese Anregung über einen kleinen Magneten auf dem Umbo, der mit einer am Kopfteil des Operationstisches angebrachten Spule angeregt wurde, verwirklicht [26]. Die resultierende Schwingung der Fußplatte wurde mit der LDV aufgezeichnet, verstärkt und über einen Kopfhörer akustisch wahrnehmbar gemacht. Ein Vergleich der günstigsten Rekonstruktion der Kette mit und ohne Real-Time-Feedback konnte für die Rekonstruktion mit PORP und mit TORP bei jeweils einem Patienten und in Felsenbeinpräparaten dargestellt werden. Es zeigte sich dabei ein deutliches Optimierungspotenzial dieser Methode bei der Kettenrekonstruktion.

Auch für Bohrarbeiten während Ohroperationen kann ein Real-Time-Feedback im Sinne eines Monitorings sinnvoll sein. Die intraoperative Messung der Schallpegel wurde von Hausold 2018 dargelegt [6]. Der Lärmeintrag wird über Knochenleitung vermittelt und kann daher auch am Knochen gemessen werden. Eine Komplettierung mit Verarbeitung der Signale bis zum Real-Time-Feedback sind bisher jedoch noch nicht vorgestellt worden. Aufgrund der bei Bohrarbeiten zu messenden Maximalschalldruckpegel bis zu 125 dB(A) [11, 22] und bis zu 130 dB(A) bei Cochleostomie [19] sowie der erheblichen Varianz während des Bohrvorgangs [6, 21] erscheint auch hierfür die Entwicklung eines geeigneten Systems als nutzbringend für die Patientensicherheit.

Nachteile eines intraoperativen Real-Time-Feedback-Verfahrens, wie Anschaffungskosten oder höherer prä- und intraoperativer Aufwand, können in ihrer Relevanz erst bei Vorliegen einsatzfähiger Systeme gegengerechnet werden. Aktuell darf vermutet werden, dass die Vorteile eines solchen Systems deutlich überwiegen und daher die weitere Entwicklung einfach einsetzbarer Systeme anzustreben ist.

## Fazit für die Praxis

- Aufgrund der hochsensiblen Strukturen innerhalb der engen Raumverhältnisse des Ohrs bestehen hohe Anforderungen an das Können eines Ohroperateurs.
- Die Komplikationsmöglichkeiten während einer Ohroperation legen die Ausbildung eines Ohroperateurs am Modell nahe.
- Tympanoplastikmodelle verbessern operationsrelevante Fertigkeiten der Übenden.
- Real-time-Feedback-Modelle in der Ohrchirurgie verbessern das Rekonstruktionsverständnis und demonstrieren schädigende intraoperative Lärmeinflüsse.
- Der zukünftige Einsatz intraoperativer Monitorsysteme zur Detektion akustischer Noxen und Dokumentation der Qualität der Kettenrekonstruktion ist naheliegend.
